# Derlin-1 overexpression confers poor prognosis in muscle invasive bladder cancer and contributes to chemoresistance and invasion through PI3K/AKT and ERK/MMP signaling

**DOI:** 10.18632/oncotarget.15001

**Published:** 2017-02-02

**Authors:** Qianze Dong, Lin Fu, Yue Zhao, Shutao Tan, Enhua Wang

**Affiliations:** ^1^ Department of Pathology, The First Affiliated Hospital and College of Basic Medical Sciences, China Medical University, Shenyang, China; ^2^ Department of Urology, Shengjing Hospital of China Medical University, Shenyang, China

**Keywords:** Derlin-1, bladder cancer, prognosis, ERK, AKT

## Abstract

Derlin-1 has been found to be overexpressed in several human cancers. However, its clinical significance and biological roles in bladder cancer remain unexplored. Here, we found that Derlin-1 was upregulated in 38.6% (58/150) cases of cancer samples. The rate of Derlin-1 overexpression was higher in muscle invasive bladder cancer (MIBC) than non-muscle invasive bladder cancer (NMIBC) (p=0.0079). Derlin-1 was a predicting factor for poor patient prognosis. Derlin-1 depletion inhibited while its overexpression facilitated cell invasion and colony formation. In addition, Derlin-1 overexpression induced cisplatin resistance while its depletion sensitized cancer cells to cisplatin. Further analysis demonstrated that Derlin-1 activated AKT phosphorylation and upregulated Bcl-2 expression. Blockage of AKT signaling by LY294005 abolished the effects of Derlin-1 on Bcl-2 and cisplatin resistance. Immunoprecipitation indicated Derlin-1 interacted with p110α subunit of PI3K. In addition, we showed that Derlin-1 depletion downregulated and its overexpression upregulated cell MMP-2/9 expression and ERK phosphorylation. Derlin-1 mediated upregulation of MMP-2/9 could be blocked by ERK inhibitor. In conclusion, our study demonstrated that Derlin-1 is overexpressed in bladder cancer and promotes malignant phenotype through ERK/MMP and PI3K/AKT/Bcl-2 signaling pathway.

## INTRODUCTION

Bladder cancer is one of the most common cancers and its incidence is increasing in recent years [1]. The prognosis of bladder cancer lies on its invasion depth, lymph node metastasis and response to chemotherapy [2–4]. Although combined therapies including surgery, radiotherapy and chemotherapy have been improved, the patient prognosis for high stage bladder cancer is still poor. Development of chemoresistance plays a vital role in the progression and poor response of bladder cancer and identifying of related targets is an important task.

Derlin-1, a partner of the p97 ATPase complex, was initially reported to mediate elimination of misfolded proteins from the endoplasmic reticulum (ER), and retro-translocation of proteins into the cytosol [5–7]. Derlin-1 can relieve ER stress-induced apoptosis in breast cancer cells [8]. Derlin-1 overexpression also ameliorates mutant superoxide dismutase 1 (SOD1)-induced ER stress and cell toxicity by reducing mutant SOD1 accumulation [9]. The role of Derlin-1 has also been implicated in human cancers. Derlin-1 expression is elevated in breast cancer and correlates with tumor grade and lymph node metastasis [8]. A study using tissue microarray showed that Derlin-1 was up-regulated in six types of human carcinomas, and Derlin-1-targeting antibodies suppressed colon tumor growth in isogenic mice [10]. Derlin-1 expression is also increased in lymph node metastases of canine mammary adenocarcinomas [11]. Our previous study demonstrated that Derlin-1 is overexpressed in non-small cell lung cancers and promotes invasion through regulation of EGFR activity [12]. These studies indicate that Derlin-1 plays an important role in cancer progression.

However, the mechanism of bladder cancer progression, especially in muscle invasive bladder cancer, has not been fully understood. The relationship between derlin-1 and bladder cancer chemoresistance also remains unclear. In this study, we examined Derlin-1 protein expression in 150 cases of bladder cancer specimens. We overexpressed and depleted Derlin-1 in bladder cancer cell lines and examined its roles on cell proliferation, invasion and chemoresistance. We also checked the molecular mechanism underlying its biological effects.

## RESULTS

### Clinical significance of Derlin-1 in human bladder cancers

**Figure 1:**
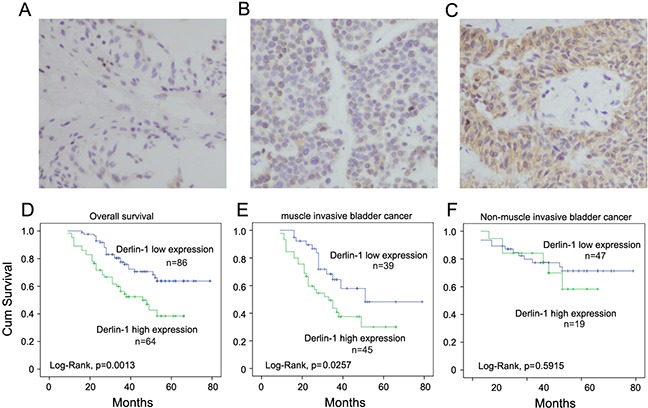
Expression pattern of Derlin-1 protein in bladder cancer tissues **A**. Negative Derlin-1 expression in normal bladder urothelial cells. **B**. Negative Derlin-1 expression in bladder cancer. **C**. Positive ctyoplasmic staining of Derlin-1 in a case of bladder cancer. **D**. Kaplan-Meier plots and Log-Rank test showed that Derlin-1 overexpression associated with poor overall survival in patients with MIBC and NMIBC. **E**. Kaplan-Meier plots of patient with MIBC. Patients with Derlin-1 overexpression showed poor survival. **F**. Kaplan-Meier plots of patient with NMIBC. Derlin-1 did not correlate with survival in patients with NMIBC. (Magnification, 400X)

We examined Derlin-1 protein expression in 150 cases of bladder cancer tissues (Figure 1). Negative Derlin-1 staining was found in normal transitional epithelial tissues (Figure 1A). Positive cytoplasmic Derlin-1 staining was observed in bladder cancer tissues. Derlin-1 overexpression was observed in 58 of 150 (38.6%) bladder cancer tissues examined. Representative examples of positive IHC staining of Derlin-1 are presented in Figure 1B and 1C. As shown in Table 1, the rate of Derlin-1 overexpression was significantly higher in muscle invasive bladder cancer (MIBC) (p=0.0079). The percentages of Derlin-1 overexpression in T2 and T3-T4 cancers were 50.9% and 58.1% respectively, which were significantly higher than that in Ta-T1 cancers. We did not find significant correlation between Derlin-1 and other clinical parameters including age, gender or tumor grade.

**Table 1:** Distribution of Derlin-1 in bladder cancer according to clinicopathological characteristics

Characteristics	Number of patients	Derlin-1 low expression	Derlin-1 high expression	*P*
Age				
<60	61	36	25	0.7301
≥60	89	50	39	
Gender				
Female	42	21	21	0.2575
Male	108	65	43	
Local invasion (T)				
Ta-T1	66	47	19	0.0079
T2	53	26	27	
T3-T4	31	13	18	
Tumor grade				
G1	73	43	30	0.7049
G2 and G3	77	43	34	

The associations between the patients’ survival and Derlin-1 were studied using Kaplan-Meier plots and Log-Rank test. As shown in Figure 1D, Derlin-1 overexpression associated with decreased overall survival (Log-Rank test, p=0.0013). Univariate analysis and multiivariate analysis (Cox model) revealed that Derlin-1 and T stage were predicting factors for poor overall survival (Table 2). We also divided these patients into 2 cohorts: those with muscle invasive bladder cancer (MIBC) and those with non-muscle invasive bladder cancer (NMIBC). Statistical analysis demonstrated that high Derlin-1expression was associated with poor prognosis in MIBC cohort (Log-Rank test, p=0.0257, Figure 1E). There was no significant association between Derlin-1 and survival in patients with non-muscle invasive bladder cancer (Figure 1F). These results indicate that Derlin-1 is overexpressed in MIBC and correlates with poor prognosis.

**Table 2:** Univariate and Multivariate analysis for predictive factors in patients with bladder cancer (Cox regression model)

	Univariate		Multivariate	
Factors	Hazard ratio(95% CI)	p value	Hazard ratio(95% CI)	p value
Age	1.293 (0.762-2.192)	0.3410	1.163 (0.675-2.006)	0.5864
Gender	1.333 (0.731-2.429)	0.3481	1.250 (0.665-2.350)	0.4876
Local invasion (T)	2.153 (1.539-3.027)	0.0001	1.979 (1.385-2.828)	0.0002
Tumor grade	1.748 (1.034-2.955)	0.0372	1.337 (0.989-1.808)	0.0591
Derlin-1	2.166 (1.293-3.630)	0.0033	1.735 (1.021-2.947)	0.0416

### Derlin-1 is overexpressed in bladder cancer cell lines and promotes proliferation and invasion

**Figure 2:**
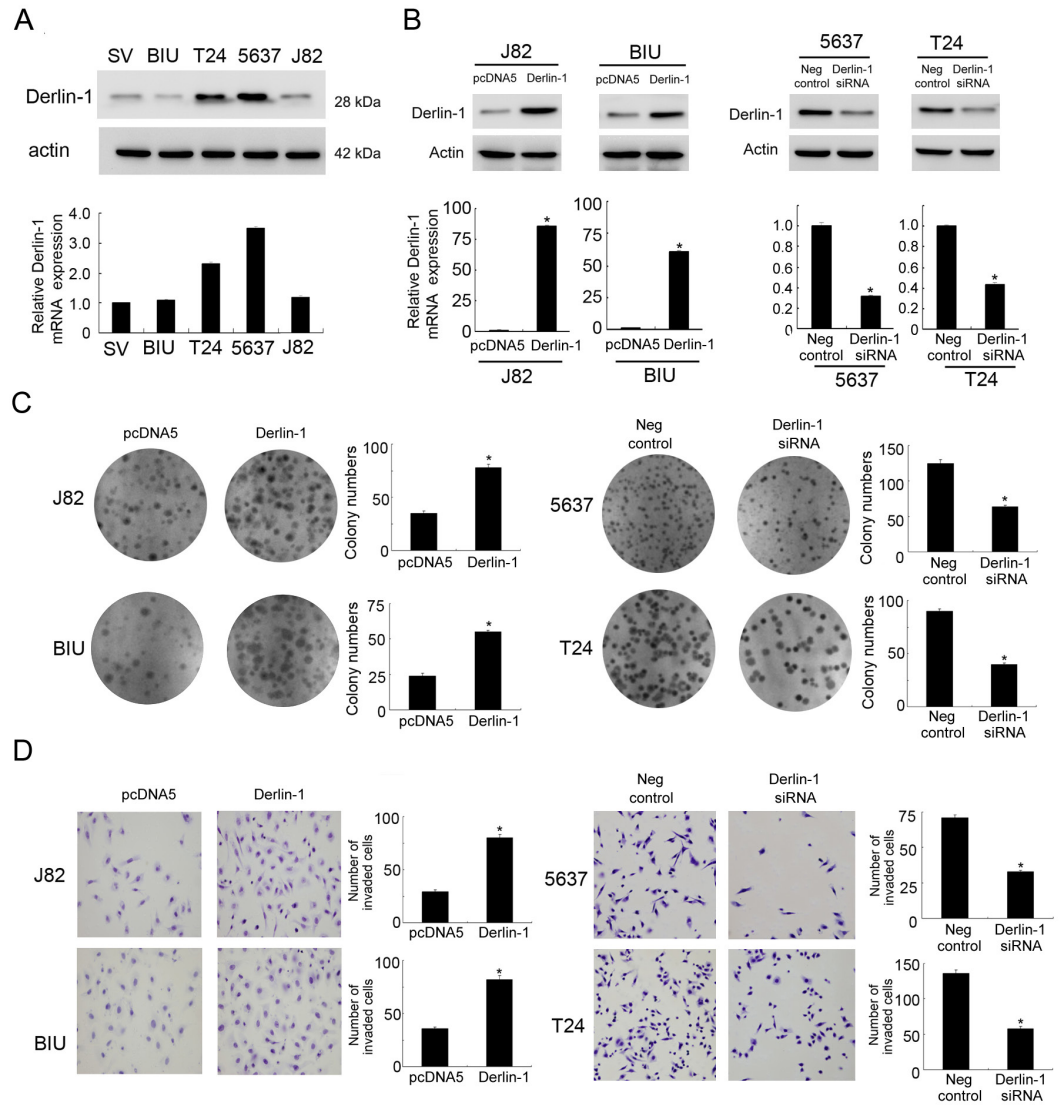
Derlin-1 overexpression in bladder cancer cell lines promotes proliferation and invasion **A**. Western blot and RT-qPCR showed that Derlin-1 was high in 5637 and T24 cell lines while low in BIU-87, J82 cancer cell lines and normal SV-HUC-1 cell line. **B**. Western blot and RT-qPCR analysis showed that Derlin-1 transfection in BIU-87 and J82 cell lines increased its protein and mRNA expression. Derlin-1 siRNA treatment in 5637 and T24 cell lines decreased its protein and mRNA expression. **C**. Colony formation assay showed Derlin-1 overexpression facilitated cell proliferation in BIU-87 and J82 cell lines, while siRNA downregulated colony number in 5637 and T24 cell lines. **D**. Matrigel invasion assay showed Derlin-1 overexpression facilitated cell invasion in BIU-87 and J82 cell lines, while siRNA downregulated invading ability in 5637 and T24 cell lines.

Endogenous expression of Derlin-1 was examined by western blot and realtime RT-PCR in normal bladder transitional epithelial cell line SV-HUC-1 and bladder cancer cell lines. We found that 5637 (MIBC) and T24 (MIBC) cell lines has high endogenous Derlin-1 expression, while BIU-87 (NMIBC), J82 cell lines and normal SV-HUC-1 cell line have low endogenous Derlin-1 expression (Figure 2A). Derlin-1 transfection and siRNA knockdown was performed in these cell lines. As shown in Figure 2B, plasmid transfection significantly upregulated Derlin-1 in J82 and BIU-87 at both protein and mRNA levels. siRNA knockdown efficiency was also confirmed in 5637 and T24 cells. Colony formation assay showed Derlin-1 overexpression facilitated cell proliferation in BIU-87 and J82 cell lines, while siRNA downregulated colony number in 5637 and T24 cell lines (Figure 2C). To characterize the role of Derlin-1 on invading ability, matrigel invasion assay was performed. As shown in Figure 2D, significant reduction of invading ability was found in cells with Derlin-1 siRNA while Derlin-1 plasmid enhanced invading ability.

### Derlin-1 regulates cisplatin resistance in bladder cancer cells

**Figure 3:**
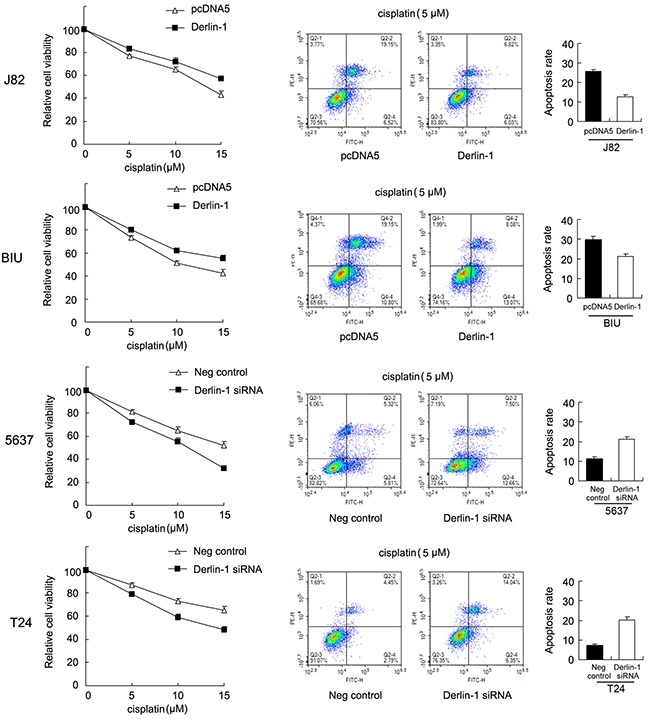
Derlin-1 confers cisplatin resistance in bladder cancer cells MTT showed that Derlin-1 overexpression significantly upregulated cell viability in BIU-87 and J82 cells after 24 hours of cisplatin treatment (5, 10, 15μM). Derlin-1 depletion downregulated cisplatin resistance in 5637 and T24 cell lines. Annexin V/PI showed that Derlin-1 overexpression significantly downregulated cell apoptosis in BIU-87 and J82 cells after 24 hours of 5μM cisplatin treatment. Derlin-1 depletion in 5637 and T24 cell lines showed the opposite effects.

To characterize the impact of Derlin-1 on drug resistance of bladder cancer cells, MTT was performed in cells treatment with different concentration of cisplatin (5, 10, 15μM, 24 hours). Annexin V/PI analysis was also carried out to examine the rate of apoptosis. As shown in Figure 3, Derlin-1 significantly upregulated cell viability in BIU-87 and J82 cells after 24 hours of cisplatin treatment. On the contrary, Derlin-1 depletion impaired cisplatin resistance in 5637 and T24 cell lines. Accordingly, a decreased percentage of cisplatin-induced apoptosis (5μM, 24 hours) was observed in J82 and BIU-87 cells transfected with Derlin-1 plasmid compared with empty vector. Derlin-1 depletion significantly upregulated cisplatin-induced apoptosis in 5637 and T24 cells, demonstrating that Derlin-1 confers chemoresistance in bladder cancer cells.

### Derlin-1 interacts with p110α and regulates chemoresistance through AKT/Bcl-2

**Figure 4:**
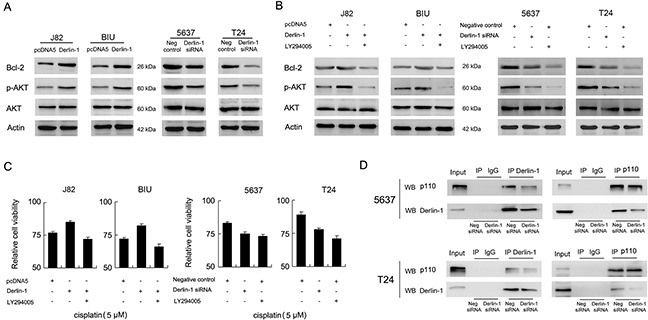
Derlin-1 interacts with p110α and regulates AKT/Bcl-2 **A**. Western blot showed thatDerlin-1 plasmid upregulated Bcl-2, p-AKT expression in BIU-87 and J82 cells. Derlin-1 siRNA downregulated Bcl-2, p-AKT expression in 5637 and T24 cell lines. **B**. Treatment of LY294005 blocked the upregulating effects of Derlin-1 on AKT phosphorylation and Bcl-2 in J82 and BIU-87 cells. LY294005 significantly downregulated p-AKT and Bcl-2 protein expression in T24 and 5637 cells. **C**. MTT results demonstrated that LY294005 reduced cell viability induced by Derlin-1 in J82 and BIU-87 cells treated with cisplatin. Similar to effects of Derlin-1 depletion, LY294005 significantly reduced cell viability in T24 and 5637 cells. **D**. Derlin-1 and PI3K p110α co-immunoprecipitated with each other in T24 and 5637 cells.

Next, we asked what is the mechanism of Derlin-1 induced chemoresistance. We checked related protein and found Derlin-1 upregulated Bcl-2, an important anti-apoptosis protein while Derlin-1 siRNA downregulated Bcl-2. Since Bcl-2 was the target of AKT signaling pathway, we checked the change of AKT activity and found that Derlin-1 positively modulate AKT phosphorylation in bladder cancer cells (Figure 4A). Next, PI3K-AKT inhibitor LY294005 was used to validate the involvement of AKT activation in Derlin-1 induced Bcl-2 and cisplatin resistance. As shown in Figure 4B, treatment of LY294005 blocked the effects of Derlin-1 on AKT phosphorylation and Bcl-2 in J82 and BIU-87 cells. Similar as Derlin-1 siRNA, LY294005 also significantly downregulated p-AKT and Bcl-2 in T24 and 5637 cells. As shown in Figure 4C, MTT results demonstrated that LY294005 reduced cell viability and chemoresistance induced by Derlin-1 overexpression. To clarify the mechanism of Derlin-1 on AKT activation, co-immunoprecipitation was performed to examine if there is an association between Derlin-1 and component of PI3K-AKT cascade. As shown in Figure 4D, Derlin-1 and PI3K p110α co-immunoprecipitated with each other in bladder cancer cells. We did not detect their association when extracts were immunoprecipitated with normal rabbit IgG or other related proteins.

### Derlin-1 regulates invasion through MMP-9 and MMP-2

**Figure 5:**
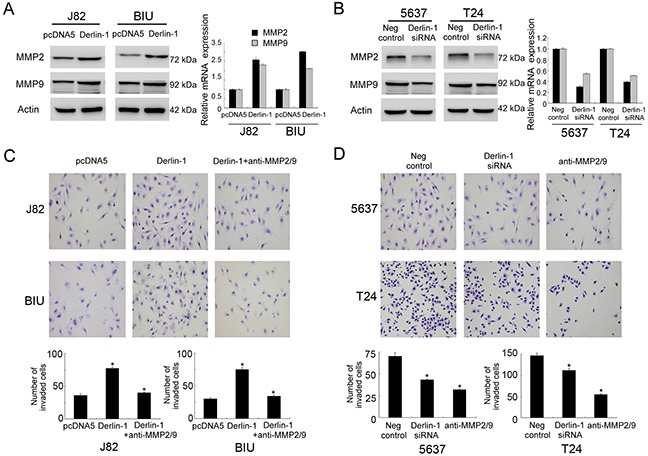
Derlin-1 regulates bladder invasion through MMP-2/9 **A**. Derlin-1 overexpression upregulated protein expression of MMP-2 and MMP-9. **B**. Derlin-1 siRNA decreased protein expression of MMP-2 and MMP-9. **C**. Matrigel invasion assay showed that treatment of MMP-2/9 antibodies blocked the invading effect of Derlin-1 in J82 and BIU-87 cell lines. **D**. Treatment of MMP-2/9 antibodies reduced invading ability of 5637 and T24 cells.

In order to investigate the potential mechanism of Derlin-1 induced bladder cancer cell invasion, we checked the expression of invasion related proteins. The results showed that Derlin-1 overexpression upregulated MMP-2 and MMP-9 (Figure 5A) while Derlin-1 siRNA decreased levels of these proteins (Figure 5B). These data suggested that Derlin-1 regulates bladder cancer invasion possibly through regulation of MMP-9 and MMP-2.

To evaluate the contribution of MMP-2 and MMP-9 in Derlin-1 induced invasion, we blocked invading ability using MMP-2 and MMP-9 neutralizing antibodies (10 μg/mL). As shown in Figure 5C, treatment of MMP-2/9 antibodies blocked the invasion promoting effect of Derlin-1 on J82 and BIU-87 cell lines. As shown in Figure 5D, treatment of MMP-2/9 antibodies reduced invading ability of 5637 and T24 cells, which was similar to the effects of Derlin-1 siRNA. Together, these results indicate that MMP-2 and MMP-9 play a central role in Derlin-1 induced invasion.

### Derlin-1 regulates MMP-2/9 through ERK signaling

**Figure 6:**
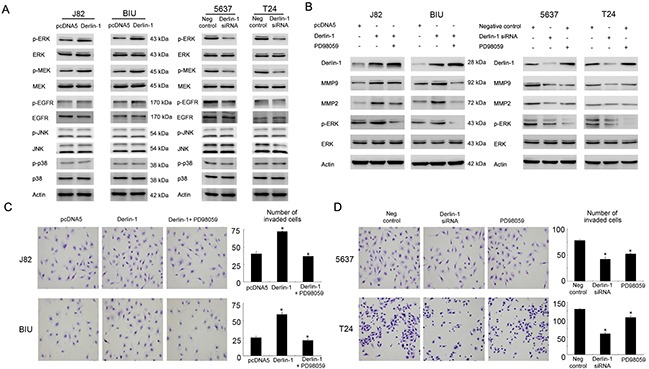
Derlin-1 regulates invasion through ERK signaling **A**. Derlin-1 overexpression significantly upregulated ERK, MEK and EGFR phosphorylation in BIU-87 and J82 cell lines, without significant changes of JNK and p38 phosphorylation. Derlin-1 depletion downregulated ERK, MEK and EGFR phosphorylation. **B**. ERK inhibitor PD98059 treatment reduced MMP-2 and MMP-9 upregulation induced by Derlin-1 in BIU-87 and J82 cells. ERK inhibitor PD98059 reduced MMP-2/9 in 5637 and T24 cell lines. **C**. ERK inhibitor PD98059 treatment downregulated invading cell number in BIU-87 and J82 cells, which was upregulated by Derlin-1 plasmid. **D**. PD98059 treatment reduced invading cells in 5637 and T24 cell lines, which is similar to the effects of Derlin-1 siRNA.

To explore the potential mechanism of Derlin-1 induced MMP-2/9. We checked MAPK signaling activities (including JNK, p38 and ERK) which were previous reported to be involved in MMP regulation. We found that Derlin-1 overexpression significantly upregulated ERK, MEK and EGFR phosphorylation. The change of JNK and p38 phosphorylation was not significant (Figure 6A). These data suggests Derlin-1 induced MEK-ERK activation may be responsible for MMP-2/9 upregulation. To confirmed this, ERK inhibitor PD98059 (20μM, 6 hours) was applied in bladder cancer cells together with Derlin-1 plasmid transfection. As shown in Figure 6B, inhibitor treatment blocked the role of Derlin-1 on MMP-2/9 upregulation in BIU-87 and J82 cells. Similar to Derlin-1 siRNA, PD98059 treatment also reduced MMP-2/9 in 5637 and T24 cell lines. In addition, we validated the effects of ERK inhibition on cell invasion. As shown in Figure 6C and 6D, ERK inhibitor treatment abolished the effect of Derlin-1 on cell invasion in BIU-87 and J82 cells and reduced invading ability of 5637 and T24 cells. These data suggest that Derlin-1 induces MMP-2/9 and cell invasion through activation of MEK-ERK pathway.

## DISCUSSION

There is evidence in the literature implicating the role of Derlin-1 in cancer progression. However, little is known about its expression pattern in bladder cancer or its role on chemoresistance. In this study, we identify elevated expression of Derlin-1 in bladder cancer tissues and cell lines, especially in MIBC. We demonstrate that Derlin-1 induces bladder cancer invasion through ERK/MMP signaling. We report for the first time that Derlin-1 contribute to the chemoresistance of cancer cells by activation of AKT signaling, with subsequent downstream Bcl-2 elevation. We further demonstrate that Derlin-1 could interact with PI3K p110α.

Immunohistochemistry showed that non-neoplastic bladder urothelium was negative for Derlin-1 protein, while Derlin-1 was upregulated in 58 of 150 bladder cancer specimens and correlated with muscle invasive phenotype (T2-T4). Importantly, we demonstrated that Derlin-1 overexpression predicted poor patient survival. Derlin-1 overexpression also correlated with poor prognosis in the MIBC cohort. In the cohort of non-muscle invasive bladder cancer (NMIBC), Derlin-1 did not exhibit significant correlation with prognosis. These results indicate that Derlin-1 signature might have clinical values in predicting and providing prognostic information for MIBC patients. Analysis of Derlin-1 in a series of cell lines showed strong Derlin-1 expression in T24 and 5637 cell lines. In contrast, normal epithelial cell line SV-HUC-1 showed very weak expression, which further supports the fact of Derlin-1 overexpression.

Then we examined the biological effects of Derlin-1 using plasmid transfection and siRNA. Our results demonstrated that Derlin-1 overexpression facilitated proliferation and invasion, while its depletion inhibited cell growth and invasion. Derlin-1 was has been reported to induce proliferation and invasion in a variety of cancers including lung and breast cancers [8, 12]. Our result is in accord with previous studies indicating Derlin-1 as a regulator of cancer aggressiveness.

The involvement of Derlin-1 in apoptosis and chemoresistance has not been well investigated before. Here, using MTT cell viability assay and AnnexinV/PI analysis, we demonstrate that Derlin-1 confers cisplatin resistance and reduced apoptosis in bladder cancer cell lines. After searching of related proteins, we found Derlin-1 positively regulated Bcl-2 and p-AKT levels while its siRNA exhibited the opposite effects. It is well established that Bcl-2 functions as an anti-apoptotic protein and a AKT target protein [13]. Bcl-2 modulates multi-drug resistance in bladder cancer cells and correlated with patient response to chemotherapy [14–16]. To determine whether Derlin-1 induced drug resistance involves PI3K/AKT activation, we examined whether treatment with the PI3K/AKT inhibitor LY294005 suppressed vialility of Derlin-1-overexpressing bladder cancer cells in response to cisplatin. Our data demonstrated that AKT inhibitor LY294005 abolished the role of Derlin-1 on Bcl-2 and chemoresistance, suggesting Derlin-1 upregulates chemoresistance and inhibits apoptosis through AKT/Bcl-2 signaling.

Since AKT signaling has been shown to be controlled by the p85 and p110 subunit of PI3K, we hypothesized that overexpression of Derlin-1 promotes its interaction with these PI3K component. We isolated Derlin-1 immune complexes from T24 and 5637 cells and detected a markedly interaction of Derlin-1 with p110α protein. Consistently, when we isolated endogenous p110α immune complex, we also detected interaction between p110α and Derlin-1. Derlin-1 siRNA decreased such interaction. To date, this is the first report regarding the mechanism of Derlin-1 in AKT activation. Our results not only reveal the involvement of Derlin-1 in AKT signaling transduction, but also provide a potential mechanism. Further studies exploring the biochemical interaction between Derlin-1 and PI3K/AKT and its signaling networks, such as the mTOR pathway, will be beneficial to understand the biological effects of Derlin-1.

Next we validate the underlying mechanism of Derlin-1 induced invasion, we examined cell cycle and invasion related proteins. We found that Derlin-1 overexpression upregulated MMP-2 and MMP-9 protein. MMP-2 and MMP-9 were reported to play important roles in bladder cancer cell invasion and metastasis [17, 18]. MMP-2/9 expression is controlled at many levels such as gene transcription, enzyme activation, and could be activated by several signaling pathways [12]. MEK-ERK signaling was reported to activate MMP-2/9 transcription [19–25]. Using MMP-2/9 antibodies and ERK inhibitor PD98059, we validate that Derlin-1 induces bladder cancer invasion through activation of ERK signaling, which in turn upregulates MMP-2 and MMP-9 protein expression. In addition, we checked EGFR status and found that Derlin-1 overexpression could upregulate EGFR phosphorylation, which was in accord with our previous findings in lung cancer [12]. Our findings link Derlin-1 with EGFR/ERK/MMP signaling and cancer invasion, which contributes to the understanding regarding mechanism of Derlin-1 in bladder cancer progression. We also noticed that PD98059 treatment could induce Derlin-1 protein in bladder cancer cells. It is reported that derlin-1 expression was enhanced by ER stress-inducing agents such as tunicamycin and thapsigargin [8]. The exact mechanism remains unknown, which needs investigation in our further research.

In conclusion, the present study demonstrates Derlin-1 is overexpressed in muscle invasive bladder cancer and correlated with aggressive behavior. Derlin-1 contributes to bladder cancer cell invasion through ERK/MMP signaling pathway. Derlin-1 also confers chemoresistance by activating AKT/Bcl-2 pathway through its interaction with p110α subunit of PI3K.

## MATERIALS AND METHODS

### Patients and specimens

The study protocol was approved by the Institutional Review Board of China Medical University. Primary tumor specimens were obtained from patients diagnosed with bladder cancer who underwent resection between 2008 and 2014 in the First Affiliated Hospital of China Medical University. Informed consents were obtained from all patients. The histological diagnosis was evaluated according to the WHO 2004 guidelines. Clinical data was obtained from medical records. Intravesical chemotherapy was carried out in patients with T1-T2 bladder cancer. Cisplatin based systemic chemotherapy was used in patients with T3-T4 bladder cancer. The first follow-up was carried out 2 months after surgery. Then cystoscopy, urine cytology and ultrasound results were evaluated every 3-6 months thereafter. The mean follow-up time was 39.3 months.

### Immunohistochemistry

4-μm tissue sections were prepared. Immunohistochemistry was performed using the Elivision plus kit purchased from Maixin (MaiXin, Fuzhou, China). Deparaffinized was performed using xylene. Sections were rehydrated with graded alcohol. Antigen retrieval was performed in citrate buffer (pH 6.0) for 2 minutes. H_2_O_2_ was used to block the endogenous peroxidase. Normal goat serum was used to reduce nonspecific binding. Then sections were incubated with Derlin-1 rabbit polyclonal antibody (1:400 dilution; Sigma) overnight at 4°C. Rabbit immunoglobulin (at the same concentration as for the antigen-specific antibody) was used as a negative control. The staining was followed by incubation with polymer secondary antibodies. The peroxidase reaction was developed with DAB plus from Maixin (Maixin, Fuzhou, China). Counterstaining was done with hematoxylin, and the sections were dehydrated in alcohol before mounting. We evaluated Derlin-1 staining according to a previous report [12]. Briefly, the cytoplasmic intensity of Derlin-1 staining was scored as 0 (no signal), 1 (moderate), 2 (strong). Percentage scores were assigned as 1- 1–25%, 2- 26–50%, 3- 51–75% and 4- 76–100%. The scores of each tumor sample were multiplied to give a final score of 0 to 8, and the tumors were finally determined as having Derlin-1 high expression (overexpression) when the tumor sample reached a score≥4; tumor samples with a score<4 were considered as having low expression.

### Cell culture and transfection

SV-HUC-1, BIU-87, J82, 5637 and 5637 cell lines were obtained from American Type Culture Collection (Manassas, VA, USA). Cells were cultured in DMEM (Invitrogen, Carlsbad, CA, USA) containing 10% FBS (Gibco, Invitrogen, USA).

The pcDNA5-DERL1 plasmid was kindly provided from Dr. Satoshi Yamashita (Department of Neurology, Faculty of Life Sciences, Kumamoto University, Japan). The plasmid was transfected into cells using Attractene Transfection reagent (Qiagen, Hilden, Germany). pcDNA5 empty vector was used as a negative control. For siRNA transfection, Derlin-1 siGENOME SMARTpool siRNA and Negative control siRNA were obtained from Dharmacon (GE healthcare, USA). DharmaFECT 1 (GE healthcare, USA) was used for siRNA transfection.

### Quantitative real-time PCR

Real-time PCR was performed using SYBR Green master mix (TAKARA, Dalian, China). PCR was performed using 7500 Real-Time PCR System (Applied Biosystems). β-actin was used as the reference gene. The relative expression of target genes were calculated using the 2^-ΔΔCt^ method. The primer sequences are as follow: Derlin-1 forward, 5′ CGACTTGAAACAGGAGCTTTTGA 3′, Derlin-1 reverse, 5′ AATCATCAGCAACTGCATATCCAT 3′; β-actin forward, 5′ ATAGCACAGCCTGGATAGCAA CGTAC 3′, β-actin reverse, 5′ CACCTTCTACAATGAGC TGCGTGTG 3′.

### Western blot analysis

Total proteins from cells were extracted in RIPA lysis buffer and quantified using the Bradford method. Protein samples were separated by SDS-PAGE and transferred to PVDF membranes (Millipore, USA) and incubated overnight at 4°C with primary antibody against Derlin-1 (1: 1000; Sigma, USA), MMP-2(ab92536), MMP-9(ab76003) (1:800, Abcam, USA), p-ERK(4370), ERK(4695), p-EGFR(3777), EGFR(2085), p-JNK(4668), JNK(9252), p-p38(4511), p38(8690), p-AKT(4060), AKT(4685), Bcl-2(4223), PI3K p110α(4249) (1:1000, Cell Signaling Technology, Boston, USA) and GAPDH (1:2000; Santa Cruz, USA). After incubation with peroxidase-coupled secondary antibody (1:2000, Santa Cruz, USA) at 37°C for two hours. Target proteins on PVDF membrane were visualized using ECL kit (Pierce) and obtained using DNR Imaging System (DNR, Israel).

### Colony formation and MTT assays

For colony formation assay, cells were transfected for 48h and plated into three 6-cm cell culture dishes (1000 cells). Cells were incubated for about 2 weeks in medium. Plates were washed with PBS and stained with Giemsa. The number of colonies with more than 50 cells was counted. The colonies were manually counted using a microscope.

For MTT assay, 24 hours after transfection, cells were plated in 96-well plates in at a concentration approximately 2000 cells per well. For quantification of cell viability, 20 μl of 5 mg/ml MTT (thiazolyl blue) solution was added to each well and incubated for 4 h at 37°C. The medium was removed from each well and 150 μl of DMSO was added to the well. The plate was measured at 490 nm.

### Matrigel invasion assay

Matrigel invasion assay was carried out using a 24-well Transwell chamber from Costar (Corning, USA) coated with 20 μl Matrigel with a dilution rate of 1:6 (BD Bioscience, USA). 48 hours after the transfection, cells were trypsinized and transferred to the upper chamber with our serum and incubated for 18 hours. Lower chamber was added with medium supplemented with 10% serum. Non-invaded cells were wiped out and cells invaded through the filter were fixed with 4% paraformaldehyde and stained with hematoxylin.

### Immunoprecipitation

Magnetic Beads (Bio-Rad SureBeads, USA) were incubated with antibodies and unbound antibodies were washed away. Then beads-antibody complex was incubated with target protein. The beads were magnetized using SureBeads magnetic rack and supernatant was discarded. Then elution buffer was used to collect purified target protein for western blot analysis.

### Statistical analysis

We adopted SPSS version 16 for statistical analysis. χ^2^ test was used to examine possible correlations between Derlin-1 expression and clinicopathologic factors. The Kaplan-Meier method was used to estimate the probability of patient survival, and differences in the survival of subgroups of patients were compared by using Mantel's log-rank test. The Cox regression model was used for multivariate analysis. Student's t-test was used to compare other date. p<0.05 was considered to indicate statistical significance.
